# Scarless circular mRNA-based CAR-T cell therapy elicits superior antitumor efficacy

**DOI:** 10.1038/s41392-025-02512-4

**Published:** 2025-12-23

**Authors:** Qinchao Hu, Hui Zhao, Kaicheng Zhou, Xuefei Tian, Qian Wang, Xianxin Hua, Xuyao Zhang

**Affiliations:** 1 Department of Biological Medicines, School of Pharmaceutical Sciences & Shanghai Engineering Research Center of Immunotherapeutics, Fudan University, Shanghai, China; 2 Byterna Therapeutics, Shanghai, China; 3 Department of Cancer Biology, Abramson Family Cancer Research Institute, Perelman School of Medicine at the University of Pennsylvania, Philadelphia, PA USA; 4 Quzhou Fudan Institute, Quzhou, Zhejiang China; 5 State Key Laboratory of Advanced Drug Formulations for Overcoming Delivery Barriers, Shanghai, China

**Keywords:** Molecular medicine, Immunotherapy, Translational research

## Abstract

Messenger RNA (mRNA)-based transient expression of chimeric antigen receptors (CARs) results in optimal safety profiles and provides promising opportunities to address existing challenges associated with viral vector-based CAR-T-cell therapies and to meet emerging medical needs for noncancerous indications. Conventional linear mRNAs, however, are intrinsically unstable and typically support short-lived protein expression, which can constrain therapeutic activity. Here, we engineered a high-efficiency permuted intron exon (PIE) platform to synthesize scarless circular mRNAs (cmRNAs) that drive robust CAR expression with extended durability. The scarless design avoids extraneous junction sequences, streamlining manufacturability and potentially reducing innate immune sensing. Compared with linear mRNAs, cmRNAs significantly increased both the magnitude and duration of anti-CD19 CAR and anti-GPRC5D CAR expression in primary human T cells. Functionally, cmRNA-based CAR-T cells elicited superior antitumor efficacy over their linear mRNA counterparts, as demonstrated by parallel lines of evidence, including in vitro antigen-specific cytotoxicity, cytokine release, and transcriptomics patterns consistent with sustained activation and absence of exhaustion signatures, as well as in vivo models demonstrating tumor elimination and prolonged survival benefits. Collectively, these findings position cmRNA as a next-generation mRNA modality for potent and controllable CAR expression, thereby providing a robust platform to unleash the full potential of mRNA technologies in cellular immunotherapy and precision medicine.

## Introduction

Engineering T cells with chimeric antigen receptors (CARs) has proven to be a landmark strategy for immunotherapy.^1^ Since 2017, several CAR-T-cell therapies have been approved by the FDA, reshaping the clinical treatment landscape of hematological malignancies. As a universal platform for highly specific elimination of targeted cells recognized by CARs, the therapeutic potential of CAR-T cells is far beyond oncology.^1,2^ Recent attempts are expanding the application frontiers of CAR-T cells to noncancerous indications such as autoimmune diseases,^3–5^ cardiac fibrosis,^6^ and aging.^7–9^

Despite these remarkable advances, currently approved CAR-T-cell therapies are continually raising major safety concerns, including serious cytokine-release syndrome (CRS), immune effector cell-associated neurotoxicity syndrome (ICANS), on-target/off-tumor toxicity, and insertion mutation-related secondary cancers.^10–16^ These side effects can be largely attributed to viral vector-based CAR engineering technologies, which inevitably lead to vector-related side effects and risks from genome integration and permanent CAR expression on T cells.^17^ Therefore, considerable efforts have been dedicated to developing alternatives, including nonviral platforms,^18^ precise CAR genome integration (e.g., CRISPR‒Cas),^19^ and mRNA-based CAR expression,^20^ to overcome the aforementioned challenges.

Among these emerging technologies, mRNA-based CAR expression has unique advantages, including transient protein expression, nonviral delivery platforms (e.g., lipid nanoparticles, LNPs), minimal risk of transgene integration, and versatile drug modalities (ex vivo mRNA CAR-T and in vivo mRNA CAR).^21–24^ Notably, the risk-benefit balance, which recapitulates the unique advantages of mRNA-based CAR-T cells in overcoming existing challenges and meeting emerging medical needs, is the utmost consideration for CAR-T-cell therapies for noncancerous indications.^1,25^ In addition, because of their simplicity, low cost, and acceptable safety profile, engineering T cells with CAR-encoding mRNAs provides an alternative strategy for off-the shell, low-cost, and safe CAR-T-cell therapy development.

Continuous efforts have been dedicated to developing both ex vivo and in vivo mRNA-based CAR-T-cell therapies over the last decade.^26–32^ Ex vivo mRNA CAR-T-cell therapies have been evaluated in early-stage clinical trials for the treatment of malignant pleural mesothelioma,^30^ pancreatic cancer,^31^ melanoma and breast cancer,^32^ and these studies collectively demonstrated the good safety and feasibility of mRNA-based CAR-T-cell therapy. Recently, the in vivo mRNA CAR therapy candidate MT-302 has entered a clinical trial (ClinicalTrials.gov: NCT05969041), representing exciting frontiers of in vivo mRNA-CAR therapies. Although mRNA-based CAR-T-cell therapies were tested in the clinic ten years ago, their clinical translation has been largely limited due to their compromised efficacy. The transient expression of mRNAs is a double-edged sword in CAR-T-cell applications. The bottleneck is that linear mRNAs are intrinsically unstable in vivo, which inevitably leads to a short duration of efficacy. Unlike linear mRNAs, circular mRNAs (cmRNAs) are covalently closed circular RNA molecules that are more stable than linear mRNAs^33,34^ and can be engineered to translate more proteins in mammalian cells.^35–37^ Conceptually, cmRNAs could prolong the translation period of CAR molecules on the surface of T cells and thus achieve longer-lasting efficacy than mRNA-based CAR-T cells do. Therefore, we hypothesized that cmRNA-based CARs could enable higher and more durable CAR expression on the cell membrane of functional immune cells than linear mRNAs, with the potential to generate more effective antitumor immunity.

Although cmRNAs serve as promising platforms for mRNA CAR-T-cell therapies, in vitro synthesis of high yields of cmRNAs with no extra sequence insertions is still challenging.^38^ For example, enzymatic ligases (such as T4 RNA ligases) are widely used circularization methods, but they are usually inefficient for long RNA chains (e.g., mRNAs) and usually suffer from extra sequence appendages during the T7-polymerase-based IVT process (which is prone to randomly appending one or more nucleotides into the 3’ end of synthesized linear mRNA).^39^ Permuted intron‒exon (PIE)-based ribozyme self-splicing strategies have advantages in terms of circularization yield and eliminate the problem of sequence inaccuracy because the ends of precursor linear mRNAs are spliced and removed from the produced cmRNAs. However, classical PIE usually inserts long sequence segments (up to 186 nt, also called scars) into produced cmRNAs, which are indispensable for triggering the circularization reaction.^35^ A recent study optimized the classical PIE system to shorten the scars to 27 nt while maintaining a comparable circularization yield.^40^ Nevertheless, those introduced scars are derived from viral PIE exons, and their unforeseeable immunogenicity raises nonnegligible risk concerns for developing cmRNA-based therapeutics.^41–43^ Recent studies have attempted to optimize PIE to synthesize scarless cmRNAs, but they still suffer from compromised circularization yields.^44,45^

Therefore, in this study, we engineered a high-yield and scarless PIE (Hi-Scarless-PIE) for synthesizing scarless cmRNA-coding CAR constructs and conducted proof-of-concept experiments to evaluate scarless cmRNA-based CAR-T cells in in vitro and in vivo models. Our findings demonstrated that scarless cmRNA-based CAR-T-cell therapy elicits superior antitumor efficacy and presents clinically relevant advantages over linear mRNA-based CAR-T-cell therapy, suggesting that scarless cmRNA-based CAR-T-cell therapy (cmCAR-T-cell therapy) could be a promising T-cell engineering platform for mRNA-based CAR-T-cell therapy.

## Results

### High-yield and scarless cmRNA synthesis via an engineered PIE

**Fig. 1:**
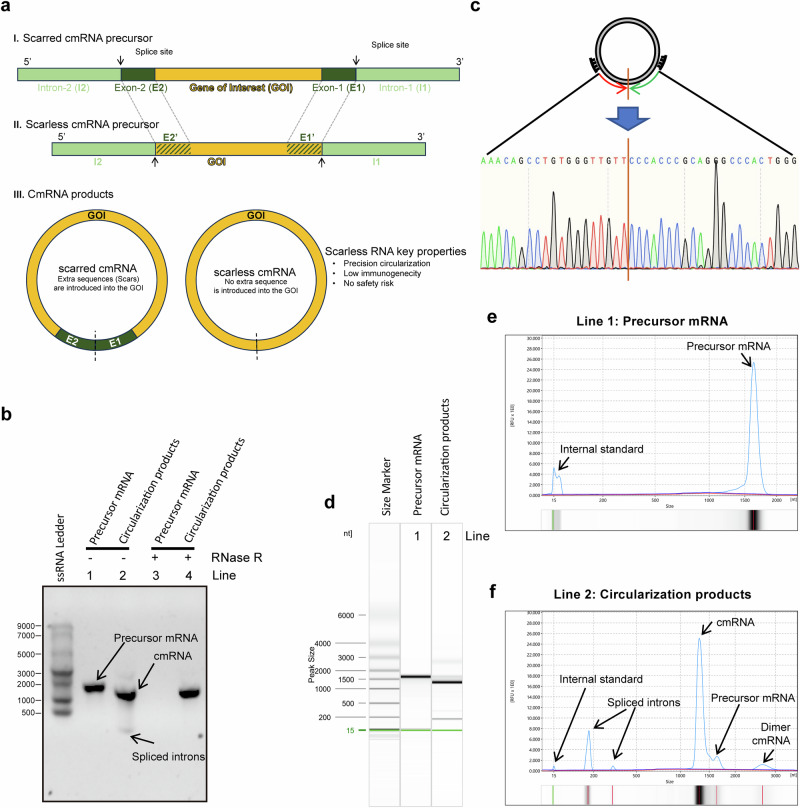
Highly efficient synthesis of scarless circRNAs via an engineered PIE-based circularization strategy. **a** Engineering strategy for scarless circular RNA synthesis. The classical PIE strategy keeps E1 and E2 residues in circular RNA products (scar circRNA), whereas the engineered new strategy generates scarless RNA products (scarless circRNA). **b** Confirmation of the generation of circular-form RNA molecules after circularization reactions via RNase R and RNase H treatments. **c** Confirmation of the precise sequence around the self-splicing junction site via Sanger sequencing. **d**–**f** Quantitative measurements of circularization reactions via capillary electrophoresis. The capillary electrophoresis separation results are shown as digital gel image bands (**d**) and classical peaks (**e**, **f**). The circularization efficiency was calculated as 100% * the area of the cmRNA peak/(the area of the cmRNA peak + the area of the precursor mRNA peak)

First, we engineered a new PIE platform based on the Anabaena tRNALeu gene with high yield and no extraneous nucleotides inserted into the cmRNA product. The rationale behind engineered PIE is to functionalize a selected 5’ end (termed E2’) and 3’ end (termed E1’) of the GOI as functions of E2 and E1 of classical PIE, respectively. As illustrated in Fig. 1a, the scarless cmRNA precursor in our engineered PIE consists of the 3’ half of the ribozyme, the GOI (which includes E2’ and E1’ surrogates at the ends of the GOI), and the 5’ half of the ribozyme. This rational design enables us to synthesize scarless cmRNA products while maintaining the high yield advantages of classical PIE.

RNase R treatment of the precursor mRNA and cmRNA products confirmed the circular format of the products after the circularization reactions, which were resistant to RNase R while the linear precursor mRNAs were digested (Fig. 1b and Supplementary Fig. 1a, b). Circular mRNA products were also confirmed by poly(A) polymerase (Supplementary Fig. 1b) and RNase H endonuclease (Supplementary Fig. 1c). Sanger sequencing revealed that the RNA sequence near the self-splicing junction site was identical to that of the designed cmRNA (Fig. 1c and Supplementary Fig. 1d), confirming the scarless ability of our engineered PIE. Capillary electrophoresis allows us to quantitatively measure the circularization yields, and the results showed that the desired cmRNA accounted for ~90% yield, which is comparable to classical PIE (Fig. 1d–f and Supplementary Fig. 1e, f). The high yield of our engineered PIE is very amenable for downstream processes such as purification and scale-up. For example, RNase R treatment was able to completely digest linear molecules and yield cmRNA with high purity (Supplementary Fig. 1g). In addition, the low immunogenicity of the yield cmRNA was confirmed by the determination of IL-6, DDX58, CCL5, TLR3, and IFNB1 in the interferon-sensitive cell line A549 (Supplementary Fig. 2). For simplicity, we named our engineered PIE Hi-Scarless-PIE (high-yield and scarless PIE) hereafter.

### Robust protein expression of synthesized scarless cmRNAs in vitro and in vivo

**Fig. 2:**
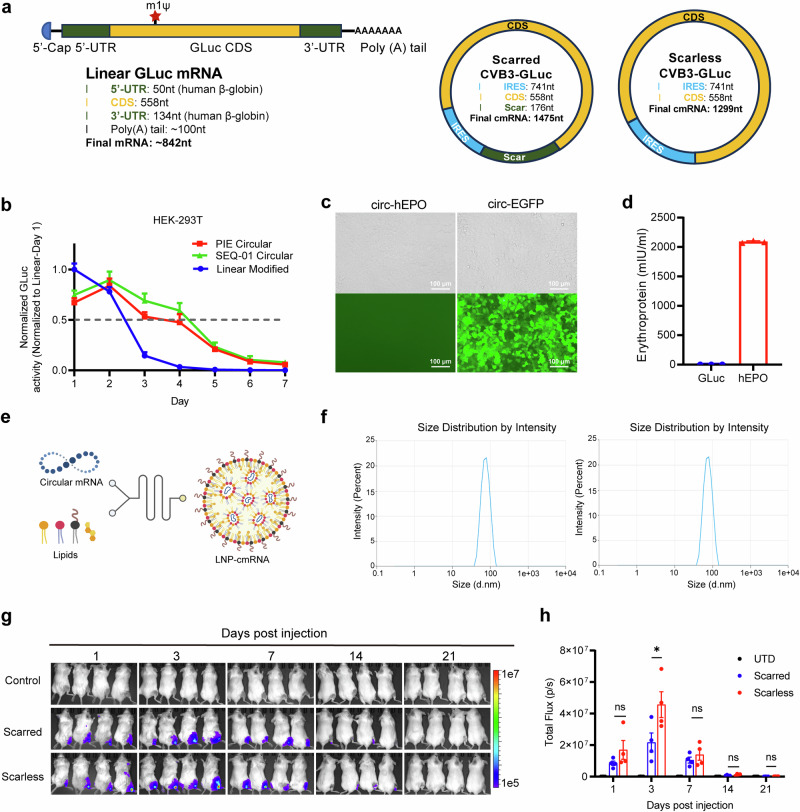
Robust protein expression of synthesized real-scarless cmRNAs in vitro and in vivo. **a** Schematic illustrations depicting the architectural configurations of linear RNA, scarred circular mRNA, and scarless circular mRNA. **b** Expression dynamics of GLuc mRNA in linear, scarred circular, and scarless circular mRNA formats in HEK-293T cells. **c**, **d** Expression of hEPO and EGFP scarless cmRNAs in HEK-293T cells was observed via fluorescence microscopy (**c**) or ELISA (**d**). **e** Illustration of the formulation process of LNPs encapsulating scarless cmRNA using a microfluidics mixer. **f** Size distributions of LNP-cmRNA particles for scar cmRNA (left) and scarless cmRNA (right). **g** The expression of GLuc cmRNA in mice after intramuscular injection of 5 µg of LNP-cmRNA per mouse was measured via bioluminescence imaging. **h** The fluorescence intensities of the mice in (**g**) are presented. The error bars indicate the SEM (two-way ANOVA; *P < 0.05; ns, not significant)

Next, we examined the translation capability of scarless cmRNA produced by Hi-Scarless-PIE in cell lines and in mice. We synthesized cmRNAs using the CVB3 IRES as the translation initiation module to translate the GLuc CDS (CVB3-GLuc). Linear GLuc mRNA (5’-capped, m1Ψ modified, and Poly(A) tailed) and scarred CVB3-GLuc cmRNA (produced by the PIE_176 nt scar version) were synthesized as controls (Fig. 2a). As shown in Fig. 2b, both scarred and scarless cmRNAs presented prolonged protein expression durations compared with those of linear mRNAs in HEK-293T cells. cmRNA-based protein expression lasted for approximately 4.5 days to reach the so-called “half-life” (here, the approximate time point at which the expression level decreased to half of the expression level on day 1), whereas the protein expression half-life of linear mRNA was approximately 2.5 days. The expression of GLuc was clearly detectable on day 7 for cmRNAs but sharply decreased on day 3 and was almost undetectable after day 4 for linear mRNAs. These results demonstrated that cmRNA driven enhanced and prolonged protein expression in vitro. Moreover, we tested the protein expression capability of scarless cmRNA produced by Hi-Scarless-PIE for other GOIs, including human erythropoietin (hEPO) and EGFP (Fig. 2c, d).

To test the in vivo protein expression of scarless cmRNAs, we formulated FLuc-coding cmRNAs with lipid nanoparticles (LNPs) via a microfluidics mixer (Fig. 2e). The formulated LNP-cmRNA exhibited an appropriate average particle diameter (~75.0 nm), PDI (<0.15), zeta potential (−5 ~ 5 mV), and encapsulation efficiency (~90%) (Fig. 2f and Supplementary Fig. 3). Intramuscular injection of 5 µg of LNP-cmRNA resulted in potent FLuc protein expression for cmRNA, and the expression lasted for 14 days (Fig. 2g). Although there was considerable interindividual variation among the mice, scarless cmRNAs consistently elicited higher FLuc expression than their scarred counterparts did on days 1, 3, and 7, with a statistically significant difference detected on day 3 (*p < 0.05) (Fig. 2h).

### Scarless cmRNAs exhibit enhanced and prolonged CAR expression in primary human T cells

**Fig. 3:**
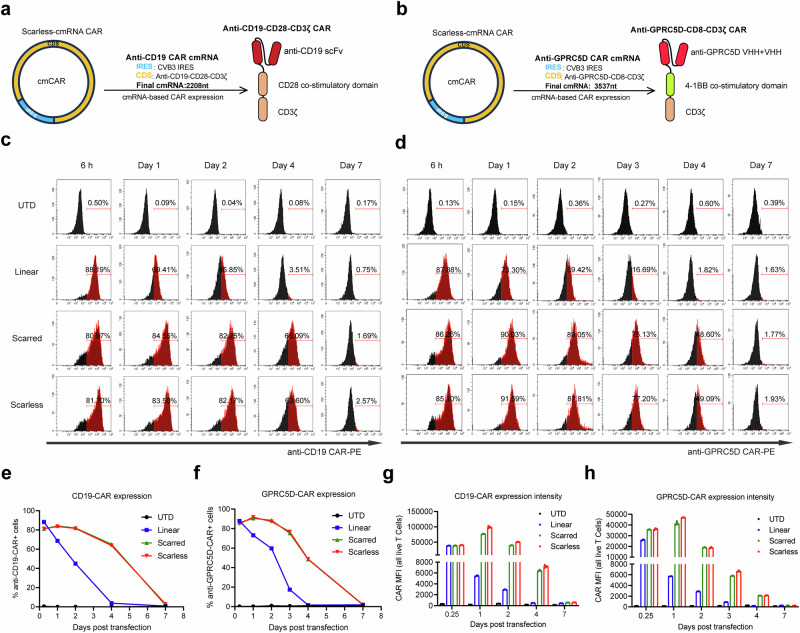
Scarless cmRNAs enhanced and prolonged the protein expression of the CD19 CAR on primary human T cells. **a**, **b** Structures of CAR-encoding cmRNA and second-generation anti-CD19 CAR (**a**) or anti-GPRC5D CAR (**b**). **c**, **d** Primary human T cells were activated and transfected with linear mRNAs (blue), scarred cmRNAs (green) or scarless cmRNAs (red) on day 4. Representative histograms of CAR expression analysis from 6 h to 7 days after transfection. PE-labeled human CD19 (20-291) protein was used for anti-CD19 CAR detection, MonoRab^TM^ Rabbits Anti-scFv Cocktail (PE) was used for anti-GPRC5D CAR detection, and Zombie Violet^TM^ Dye was used to exclude dead cells. **e**, **f** Proportion of CAR-expressing cells indicated by anti-CD19 CAR^+^ cells (E) and anti-GPRC5D CAR^+^ cells (F) from 6 h to 7 days post-transfection (*n* = 3 independent experiments/donors). **g**, **h** The median intensity of CAR expression from 6 h to 7 days post-transfection (*n* = 3 independent experiments)

To compare the translational potential of circular versus linear mRNAs for CAR-T-cell engineering, we generated anti-CD19 CAR-T cells and anti-GPRC5D CAR-T cells by electroporating primary human T cells with either linear mRNAs or cmRNAs (Fig. 3a, b). The surface expression of the CARs was assessed via the use of PE-labeled human CD19 (20-291) protein or MonoRab^TM^ rabbit anti-scFv cocktail. In the linear mRNA group, CAR expression peaked within 6 h post-electroporation and began to decrease, reaching undetectable levels by the fourth day post-transfection. In contrast, cmRNA-driven CAR expression peaked one day after transfection and was sustained for two days; thereafter, it began to decrease, with undetectable levels measured by the seventh day post-transfection. These data suggested that cmRNAs confer a more sustained expression profile in T cells (Fig. 3c–f and Supplementary Fig. 4a, b). Moreover, quantitative analysis of fluorescence intensity further revealed that cmRNA-transfected T cells presented a greater median fluorescence intensity (MFI) at peak expression than the linear mRNA group did, indicating increased CAR density on the T-cell surface (Fig. 3g, h). These findings suggest a greater density of CAR-T cells within the circular cohort than in the linear cohort, suggesting that the CAR-T cells in the circular groups presented increased cytotoxic potential (Fig. 3g, h). Consistent with these findings, RT‒qPCR analysis demonstrated that cmRNA exhibited greater stability and a significantly extended intracellular half-life relative to its linear counterpart (Supplementary Fig. 4c, d). In summary, the aforementioned results establish scarless cmRNAs as a potent platform for achieving sustained and high-level CAR expression in primary human T cells, with clear implications for improving the performance of mRNA-based cell therapies.

### cmCAR-T cells demonstrate potent cell-killing activities, specifically against malignant cells

**Fig. 4:**
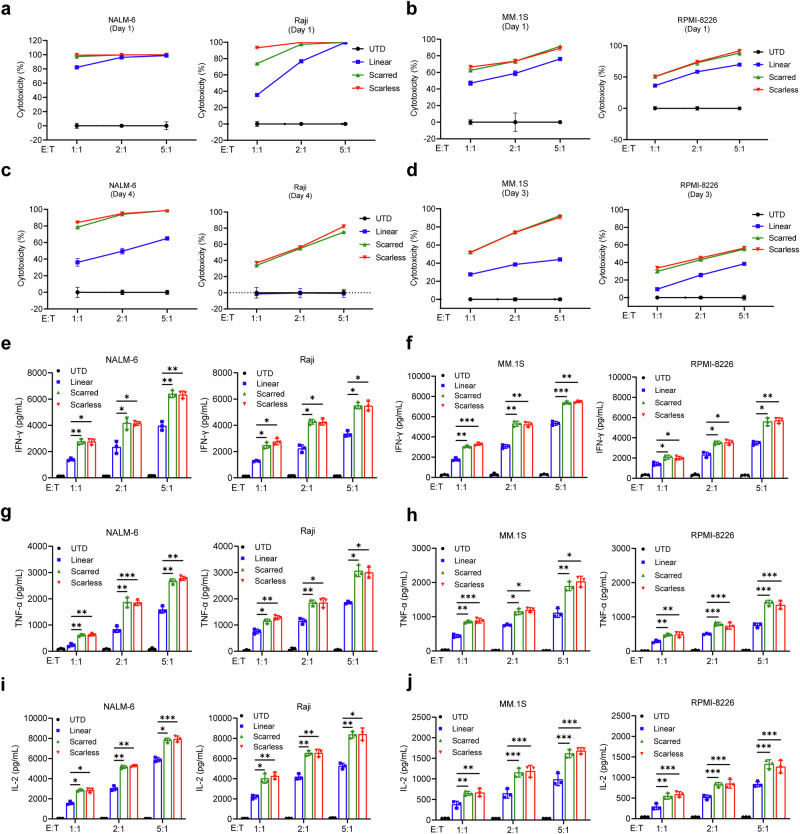
cmCAR-T cells exhibited potent target cell-killing activities with increased cytokine release. **a**, **b** One day after transfection, anti-CD19 CAR-T cells generated from linear mRNAs, scarred cmRNAs, or scarless cmRNAs were cocultured with NALM-6 or Raji cells (**a**), and anti-GPRC5D CAR-T cells from the same mRNA platforms were cocultured with MM.1S or RPMI-8226 cells (**b**). **c**, **d** Four days after transfection, anti-CD19 CAR-T cells generated from linear mRNAs, scarred cmRNAs, or scarless cmRNAs were cocultured with NALM-6 or Raji cells (**c**), and anti-GPRC5D CAR-T cells from the same mRNA platforms were cocultured with MM.1S or RPMI-8226 cells (**d**). All cocultures were conducted at effector-to-target (E:T) ratios of 1:1, 2:1, and 5:1 for 16 h. **e**–**j** IFN-γ release (**e**), TNF-ɑ release (**g**), and IL-2 release (**I**) by linear mRNA-, scarred cmRNA- or scarless cmRNA-based anti-CD19 CAR-T cells after 16 h of coculture with NALM-6 or Raji cells. IFN-γ release (**f**), TNF-α release (**h**), and IL-2 release (**j**) by linear mRNA-based, scarred cmRNA-based or scarless cmRNA-based anti-GPRC5D cells after 16 h of coculture with MM.1S or RPMI-8226 cells. Untransfected (UTD) T cells were used as a negative control. The data are presented as the means ± SDs (*n* = 3 independent experiments; two-way ANOVA; *P < 0.05; **P < 0.01; ***P < 0.001; ns, not significant)

We next evaluated the in vitro cytotoxicity of linear mRNA-based CAR-T (mCAR-T) and cmRNA-based CAR-T (cmCAR-T) cells via coculture assays with relevant tumor targets. For anti-CD19 CAR-T cells, CD19-positive NALM-6 and Raji cells, as well as CD19-negative K562 cells, were used as target populations (Supplementary Fig. 5a). For anti-GPRC5D CAR-T cells, we selected GPRC5D-positive MM.1S and RPMI-8226 cells, alongside GPRC5D-negative K562 cells (Supplementary Fig. 5b). CAR-T cells were cocultured with target cells at effector-to-target (E:T) ratios of 1:1, 2:1, and 5:1 for 16 h. Untransfected T cells were used as negative controls. The strengths and variations of the three types of CAR-T cells were assessed via flow cytometry. In the context of anti-CD19 CAR-T cells, both CAR-T-cell variants induced near-complete lysis of CD19-positive neoplastic cells while sparing their CD19-negative counterparts under diverse coculture ratios on the initial day following transfection (Fig. 4a and Supplementary Fig. 6a). By the fourth day post-transfection, the cytolytic activity of the mCAR-T cells had declined substantially. The killing efficiency of mCAR-T cells against NALM-6 cells decreased to 40–60%, and these cells failed to elicit measurable cytotoxicity against Raji cells. In contrast, the cmCAR-T cells maintained robust activity, eliminating 75–100% of the NALM-6 cells and 35–80% of the Raji cells (Fig. 4c). A similar pattern was observed with anti-GPRC5D CAR-T cells. While both CAR-T-cell variants were effective against MM.1S and RPMI-8226 cells on day 1, only cmCAR-T cells retained strong cytotoxic function by day 3, resulting in 50–95% lysis of MM.1S cells and 35–55% lysis of RPMI-8226 cells. mCAR-T cells showed markedly reduced activity, with 25–45% lysis of MM.1S and 10–35% lysis of RPMI-8226 cells (Fig. 4b, d and Supplementary Fig. 6b). Collectively, these outcomes substantiated the superior cytotoxic potency of cmRNA-based CAR-T cells against antigen-positive malignant cells, in line with their enhanced expression kinetics and stability.

### cmCAR-T cells elicit enhanced cytokine responses upon antigen engagement

To further evaluate effector function, we quantified the secretion of key cytokines, including interferon gamma (IFN-γ) and tumor necrosis factor alpha (TNF-α), which are critical for T-cell-mediated cytotoxicity, as well as interleukin-2 (IL-2), a marker of CAR-T-cell activation and persistence following the coculture of CAR-T cells with target tumor cells. As shown in Fig. 4e–j, untransfected T cells did not release detectable levels of the aforementioned cytokines when exposed to either CD19- or GPRC5D-positive tumor cells. Similarly, both mCAR-T cells and cmCAR-T cells failed to produce cytokines in response to antigen-negative targets, confirming the antigen specificity of the response (Supplementary Fig. 6c, d). Upon stimulation with antigen-positive tumor cells, both linear mRNA- and cmRNA-based CAR-T cells exhibited dose-dependent cytokine production. Notably, the cmRNA-based CAR-T cells produced significantly higher levels of IFN-γ, TNF-α, and IL-2 than did their linear mRNA counterparts across all tested E:T ratios (Fig. 4e–j). This trend was observed for both anti-CD19 CAR-T cells (Fig. 4e, g, i) and anti-GPRC5D CAR-T cells (Fig. 4f, h, j), in accordance with their enhanced cytolytic activity.

### Dynamic changes in the transcriptomic profiles of mCAR-T cells and cmCAR-T cells over time

**Fig. 5:**
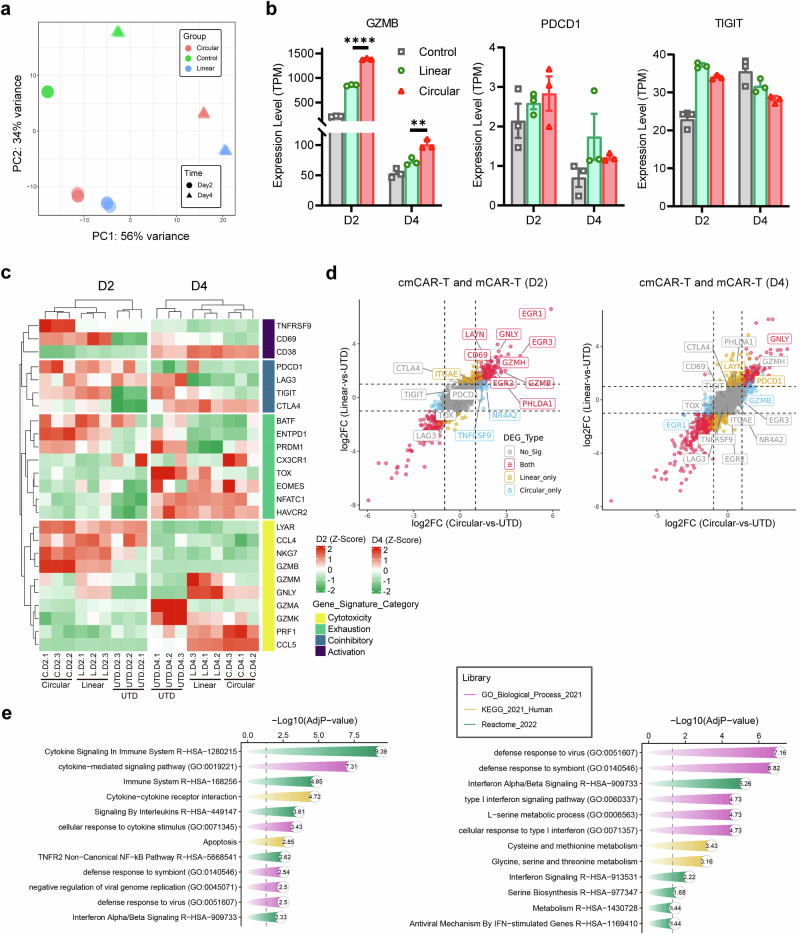
Transcriptomic profiles of mCAR-T and cmCAR-T cells on days 2 and 4 posttransfection. **a** Distribution of all samples under PCA dimensionality reduction analysis. **b** The expression levels (TPMs) of the GZMB and PDCD1 genes across groups. **c** The expression profiles of T-cell activation, coinhibitory, exhaustion, and cytotoxic gene signatures were visualized via a heatmap. **d** Differentially expressed genes (DEGs) between cmCAR-T cells and UTDs and DEGs between mCAR-T cells and UTDs were projected on the x-axis and y-axis, respectively, according to their log2FC values. Representative genes involved in T-cell activation, exhaustion, and cytotoxicity are labeled with gene names. **e** Enriched biological processes and signaling pathways of upregulated DEGs in cmCAR-T cells compared with mCAR-T cells on days 2 and 4 posttransfection (**P < 0.01, ****P < 0.0001)

Several studies have revealed that CAR-T cells produced in different ways^46^ and CAR-T cells in patients with different outcomes (e.g., responders and long-term remission patients) have different transcriptomic profiles.^47–49^ Therefore, we conducted RNA-seq experiments in an attempt to understand the differences in cell killing and cytokine release behaviors between mCAR-T cells and cmCAR-T cells from a transcriptional perspective. Overall, untransfected T cells, cmCAR-T cells, and mCAR-T cells were distinctly different from each other on days 2 and 4, as indicated by the PCA dimension reduction analysis at the transcriptomic level shown in Fig. 5a. The GZMB gene expression level in cmCAR-T cells was significantly greater than that in mCAR-T cells on both day 2 and day 4 (Fig. 5b), which is in line with our previous results that cmCAR-T cells showed greater cytotoxic activity than mCAR-T cells did (Fig. 4). The representative coinhibitory genes PDCD1, TIGIT, LAG3 and CTLA4 presented different expression levels and changes (Fig. 5b and Supplementary Fig. 7). The absolute expression levels of PDCD1 (0.2–3.5 TPM) in all groups were much lower than those of TIGIT (20–40 TPM). Transfection of the mCAR or cmCAR resulted in increased expression of TIGIT on day 2 but decreased expression on day 4 compared with the control baseline (Supplementary Table 2). This finding might suggest that different transcriptomic profiles of CAR-T functional gene signatures differ between mCAR-T cells and cmCAR-T cells at different time points.

Therefore, we visualized well-documented gene signatures related to T-cell activation, coinhibitory signals, T-cell exhaustion, and T-cell cytotoxicity via a heatmap (Fig. 5c). The dendrogram at the top of the heatmap shows that the control T-cell samples, mCAR-T-cell samples, and cmCAR-T-cell samples were clearly clustered into 3 clusters according to the heatmap hierarchical clustering algorithm, indicating obviously different overall patterns of these gene signatures between mCAR-T- and cmCAR-T-cell populations. To visualize the distribution of these gene signatures across the whole transcriptomic landscape, we performed DEG analysis and plotted all genes according to their log2FC values in both the mCAR-T-vs-Control and cmCAR-T-vs-Control DEG analyses. As shown in Fig. 5d, on day 2, 236 significantly upregulated DEGs were detected in both mCAR-T cells and cmCAR-T cells, including those encoding cytotoxic genes (GZMB, GZMH) and T-cell activation markers (CD69, GNLY, EGR1, EGR2, EGR3), whereas many T-cell exhaustion and coinhibitory signaling genes (TIGIT, CTLA4, PDCD1, TOX, and LAG3) did not significantly differ between mCAR-T cells and cmCAR-T cells compared with control T cells, except for PHLDA1 and LAYN. However, many of those T-cell activation- and cytotoxicity-related genes did not reach the DEG threshold on day 4, except that LAYN (a T-cell exhaustion marker) was significantly upregulated only in mCAR-T cells. To further understand the differences in transcription between mCAR-T cells and cmCAR-T cells, we directly compared cmCAR-T cells with mCAR-T cells via DEG analysis. There were 113 and 232 upregulated DEGs in cmCAR-T cells on days 2 and 4, respectively. Enrichment analysis revealed that these DEGs were significantly overrepresented in several cytokine signaling pathways on day 2 and interferon signaling on day 4 in cmCAR-T cells (Fig. 5e), which is in agreement with previous data (Fig. 4e–j and Supplementary Table 2). Moreover, as shown in Supplementary Fig. 8, prolonged CAR expression may induce transcriptomic differences between mCAR-T cells and cmCAR-T cells, including alterations in transcriptional programs; innate immune sensing of RNA constructs; and differences in metabolic, memory-related, and translational features.

Collectively, these findings suggest that T-cell activation and cytotoxic signals might differ between mCAR-T cells and cmCAR-T cells and that CAR-T functional signals are transient in both mCAR-T cells and cmCAR-T cells; however, cmCAR-T cells exhibit enhanced and prolonged cytotoxicity compared with mCAR-T cells.

### Anti-CD19 CAR-T cells eliminate NALM-6 cells in a mouse model

**Fig. 6:**
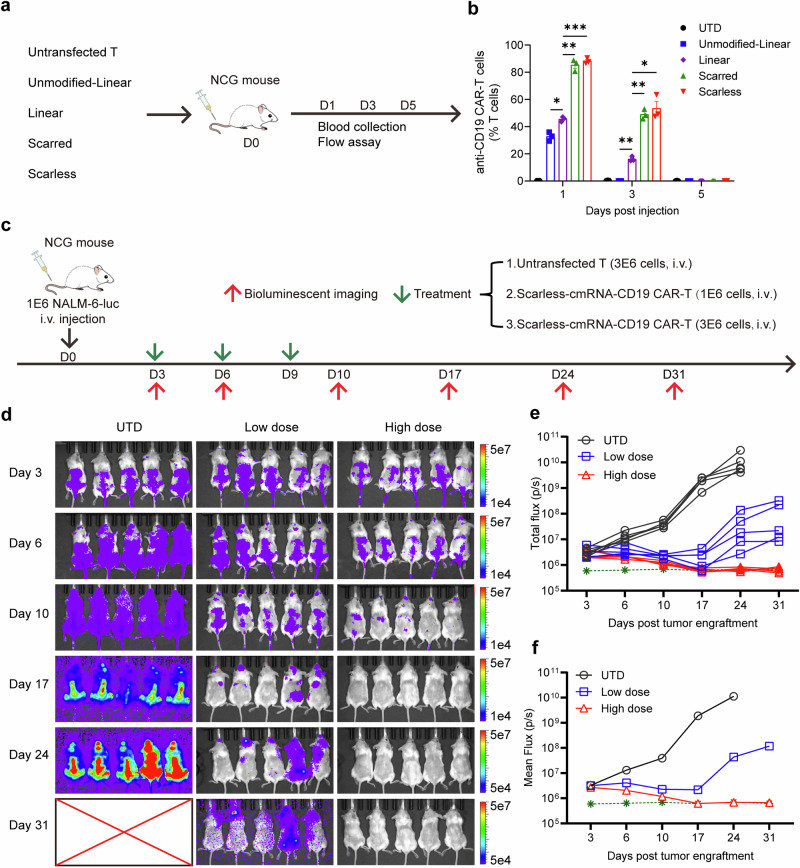
Anti-CD19 CAR-T cells eliminated NALM-6 cells in vivo. **a**, **b** Schematic diagram and detection of the persistence of CAR-T cells in mice via flow cytometry; *n* = 3 mice per group. **c** Schematic diagram of the NALM-6 tumor model and procedure of scarless cmRNA-based anti-CD19 CAR-T-cell infusion time and dose. **d** Bioluminescence imaging analysis of NALM-6 tumors; *n* = 5 mice per group. **e**, **f** The tumor burden was quantified as the total flux (each line represents a single mouse) or mean flux from the luciferase intensity of each mouse, and the green dashed line represents the background value. The data are presented as the means ± SEMs (two-way ANOVA; *P < 0.05; **P < 0.01; ***P < 0.001)

To further investigate the in vivo persistence of mCAR-T and cmCAR-T cells, we administered CAR-T cells one day post-electroporation via tail vein injection at a dose of 3×10^6^ cells per mouse (Fig. 6a). Blood samples were collected on day 1, 3, and 5 postinjection to assess the duration of CAR-T-cell presence within the mice. As depicted in Fig. 6b, CAR-T cells were detectable on the first day post-injection, with the unmodified and modified linear mRNA groups constituting 32% and 47% of the total number of circulating T cells, respectively, while the number of T cells in the circular mRNA groups surpassed 80%. By the third day post-injection, unmodified linear mRNA-based CAR-T cells were undetectable, the percentage of modified linear mRNA-based CAR-T cells had decreased to 18%, whereas the percentage of circular mRNA-based CAR-T cells had remained greater than 50%. These findings are in accordance with previous in vitro results, indicating that cmRNA-based anti-CD19 CAR-T cells exhibited prolonged persistence in vivo. We subsequently evaluated the therapeutic timing and dosage for the cmRNA group. As shown in Fig. 6c, 1 × 10^6^ luciferase-labeled NALM-6 (NALM-6-luc) cells were injected into each mouse on day 0. Tumor proliferation within the mice was monitored through bioluminescence imaging, with imaging conducted on the third day, indicating the commencement of treatment. The low-dose and high-dose groups received injections of 1 × 10^6^ and 3 × 10^6^ cmRNA-based anti-CD19 CAR-T cells per mouse, respectively. The treatment was conducted every four days three times. Bioluminescence imaging results indicated that the tumor burden in both the low-dose and high-dose treatment cohorts was significantly lower than that in the untransfected group. Following three rounds of treatment, a trend toward increased tumor proliferation was observed within the low-dose group, with one particular instance of growth noted. Moreover, high-dose treatment resulted in more efficacious tumor clearance than did low-dose treatment (Fig. 6d–f). These findings suggest that a dosage regimen of 3 × 10^6^ CAR-T cells coupled with an increased frequency of treatment may be warranted.

### cmRNA-based CAR-T cells elicit potent antitumor efficacy in vivo

**Fig. 7:**
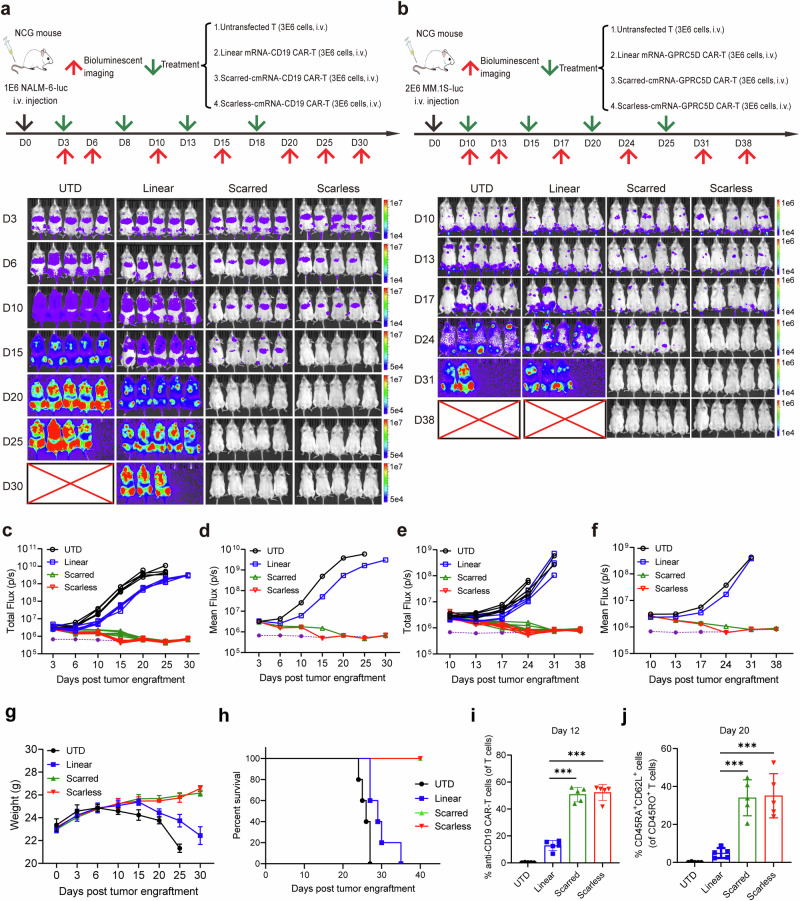
cmCAR-T cells outperform mCAR-T cells in eliminating target cells in vivo. **a**, **b** Schematic diagram of the NALM-6 tumor model and procedure of linear mRNA-, scarred cmRNA- or scarless cmRNA-based anti-CD19 CAR-T-cell infusion time and dose (**a**). Schematic diagram of the MM.1S tumor model and procedure of linear mRNA-, scarred cmRNA- or scarless cmRNA-based anti-GPRC5D CAR-T-cell infusion time and dose (**b**). The tumor burden was monitored via bioluminescence imaging analysis; *n* = 5 mice per group. **c**, **d** NALM-6 tumor burden was quantified as the total flux (**c**, each line represents a single mouse) or mean flux (**d**) from the luciferase intensity of each mouse. The purple dashed line represents the background value. **e**, **f** MM.1S tumor burden was quantified as the total flux (E, each line represents a single mouse) or mean flux (**f**) from the luciferase intensity of each mouse. The purple dashed line represents the background value. **g**, **h** Body weight change curves (**g**) and survival curve analysis (**h**) of the mice in the different treatment groups in the NALM-6 tumor model. **i** On the twelfth day of the experiment (**a**), the proportion of anti-CD19 CAR-T cells was determined by flow cytometry. **j** On the twentieth day of the experiment (**a**), the proportion of memory T cells was assessed by flow cytometry (***P < 0.001)

We next assessed the feasibility and efficacy of cmRNA-based CAR-T cells in xenograft models of B-cell lymphoma and multiple myeloma. In a B-cell lymphoma model, NCG mice were intravenously inoculated with 1 × 10⁶ NALM-6-luc cells on day 0, followed by infusion of 3 × 10⁶ untransfected T cells, linear mRNA-, scarred cmRNA- or scarless cmRNA-based anti-CD19 CAR-T cells on day 3 (Fig. 7a). Longitudinal bioluminescence imaging revealed that, compared with control mice, cmRNA-based CAR-T cells significantly reduced the tumor burden (Fig. 7c, d). A similar trend was observed in the multiple myeloma model, in which 2 × 10⁶ MM.1S-luc cells were engrafted and treated with anti-GPRC5D CAR-T cells (Fig. 7b). Again, cmRNA-based CAR-T cells demonstrated superior tumor control over linear mRNA-transfected T cells and untransfected controls (Fig. 7e, f). Moreover, significant body weight loss was observed in the control groups due to the severe tumor burden, whereas a relatively stable body weight was observed in the groups infused with cmRNA-based CAR-T cells (Fig. 7g). Notably, cmRNA-based CAR-T-cell treatment markedly prolonged survival compared with that of the other groups (Fig. 7h). On day 12, peripheral blood samples were collected to detect CAR-T cells, and the data revealed a significantly greater frequency of CAR-T cells in the cmRNA groups than in the linear mRNA cohort (Fig. 7i and Supplementary Fig. 9). By day 20, cmRNA-treated mice also presented an increased proportion of memory T cells, indicating enhanced persistence and functional longevity of the engineered T cells (Fig. 7j and Supplementary Fig. 9, Fig. 10).

**Fig. 8:**
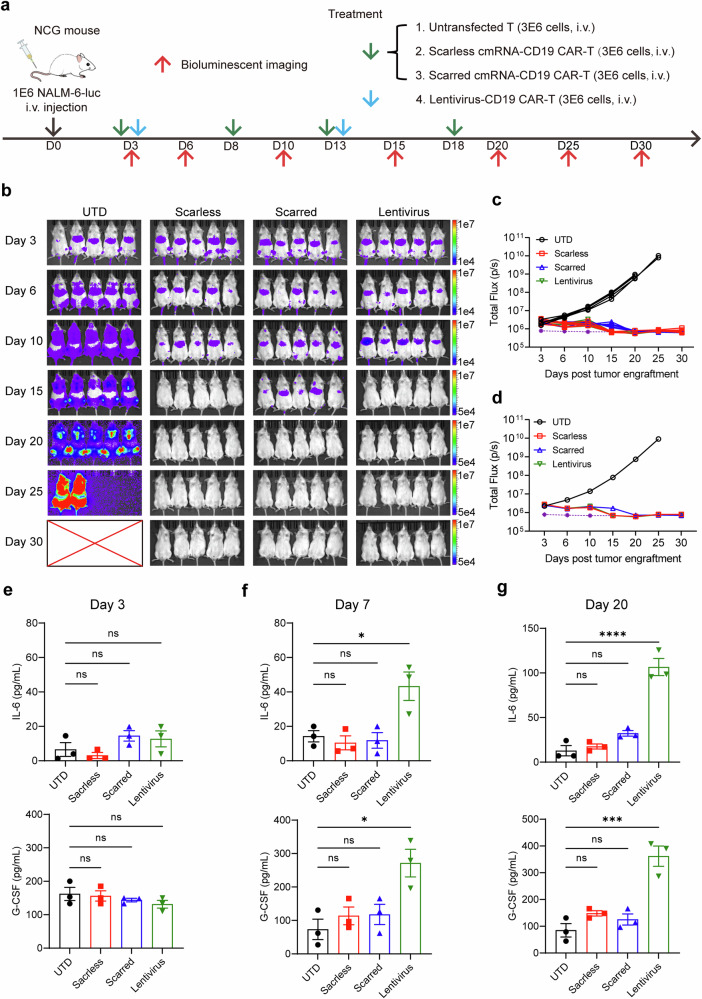
cmCAR-T cells achieved antitumor effects similar to those of lentivirus-transduced CAR-T cells, with the potential to mitigate adverse side effects. **a** Schematic diagram of the NALM-6 tumor model and procedure of scarless cmRNA-based, scarred cmRNA-based or lentivirus-transduced anti-CD19 CAR-T-cell infusion time and dose. **b**–**d** Bioluminescence imaging analysis was used to monitor the NALM-6 tumor burden in the different groups (**b**). The tumor burden was quantified as the total flux (**c**, each line represents a single mouse) or mean flux (**d**) from the luciferase intensity of each mouse. The purple dashed line represents the background value. **e**–**g** The serum levels of IL-6 and G-CSF in the different groups were measured via ELISA on the third (**e**), seventh (**f**) and twentieth (**g**) days after CAR-T-cell infusion. The data are presented as the means ± SEMs (*n* = 3; one-way ANOVA; *P < 0.05; ***P < 0.001; ****P < 0.0001; ns, not significant)

To benchmark against a clinical standard, we compared scarred cmRNA- and scarless cmRNA-based anti-CD19 CAR-T cells with lentivirus-transduced anti-CD19 CAR-T cells. Both cell types were administered in parallel in the NALM-6 model (Fig. 8a). Bioluminescence imaging demonstrated that cmRNA-CAR-T cells achieved tumor clearance comparable to that of lentiviral CAR-T cells (Fig. 8b–d and Supplementary Fig. 11), supporting the therapeutic equivalence of this nonviral approach. We further assessed CRS by monitoring IL-6, G-CSF, and CRP levels on days 3, 7, and 20 postinfusion. As shown in Fig. 8e–g and Supplementary Fig. 12, no overt CRS symptoms or significant elevations in CRP were observed in any group, likely due to the relatively low initial tumor burden. However, IL-6 and G-CSF levels were significantly elevated in the lentiviral CAR-T-cell group but not in the scarred and scarless cmRNA cohorts, suggesting that cmRNA-based CAR-T-cell therapy may mitigate the inflammatory toxicity associated with viral vector-based therapies. To ensure parity with the lentiviral setting, we also conducted single-infusion studies (Supplementary Fig. 13). In this context, lentiviral CAR-T cells provided the most durable tumor control, yet both scarred and scarless cmRNA-based CAR-T cells significantly outperformed linear CAR-T cells, confirming the advantage of the circular format even after a single dose. Notably, scarless cmRNA-based CAR-T cells maintained tumor suppression longer than their scarred counterparts did, resulting in increased survival on day 35. Collectively, these results demonstrated that cmRNA-based CAR-T cells mediate potent and durable antitumor activity in vivo, with functional persistence and a favorable safety profile, establishing cmRNAs as promising nonviral platforms for CAR-T-cell engineering.

## Discussion

Several CAR-T-cell therapies have been approved for hematological malignancies, and ongoing investigations are expanding CAR-T-cell therapy to solid tumors and other indications beyond cancer.^1,2^ However, extensive clinical applications have raised a series of safety concerns, including the risk for secondary T-cell malignancies following CD19-directed or BCMA-directed CAR-T-cell therapies, which may result from inaccurate transgene integration of the CAR. The FDA explicitly stated that, with all gene therapy products using integrating vectors, the potential risk of developing secondary malignancies is categorized as a class warning.^50^ In addition to the dangerous safety risk, viral vector-based manufacturing of CAR-T cells has a relatively high incidence of side effects such as CRS. A safer version of CAR-T cells is urgently needed to minimize safety issues and, more importantly, unleash the full potential of CAR-T-cell therapy for noncancerous indications, which is extremely sensitive to risk‒benefit balance.^1,11,17^

The use of an mRNA platform is an ideal strategy to avoid the risks associated with viral vectors, genome integration, and permanent transgene expression associated with traditional CAR-T-cell technologies.^21^ Indeed, ex vivo mRNA-based CAR-T cells have been tested for an extended period in early-stage clinical trials. Along with advances in targeted LNP delivery systems, in vivo CAR-T-cell therapy, which generates CAR-T cells in the human body via targeted delivery of CAR-encoding mRNAs into T cells, will be a gamechanger in the development of the next generation of CAR-T-cell therapies.^23,24^ However, the intrinsically unstable nature of linear mRNAs limits the efficacy of mRNA-based CAR-T cells because of the short duration of CAR expression. Recent advances in mRNA platform technology have emphasized control over RNA architecture and chemical modification. For instance, self-amplifying RNAs can transiently boost protein expression, while they remain challenges in innate immune activation and control of replication.^44^ By contrast, circular RNAs, owing to their covalently closed topology, are intrinsically resistant to exonuclease‑mediated decay and can sustain translation for prolonged periods without replication or genomic integration. These properties make cmRNAs particularly appealing for applications requiring controlled, repeatable protein expression, such as in vivo CAR‑T cell generation or transient immunomodulation.^23^ Moreover, the capacity to tune expression kinetics through sequence and structural design may enable cmRNAs to approximate physiological T‑cell activation dynamics, thereby mitigating exhaustion and enhancing persistence.

Since the first report of highly efficient in vitro synthesis of cmRNA in 2018,^35^ circular RNA has gained much attention as an updated mRNA platform for therapeutic purposes.^37,40,51^ In this study, we developed a new PIE strategy to synthesize cmRNAs with scarless and high-yield capabilities, which are very important drugability considerations for drug development. We confirmed that cmRNA increases the amount and extends the duration of CAR expression on human T cells. Using an approved anti-CD19 CAR construct as an example, we subsequently demonstrated the in vitro and in vivo advantages of cmRNA for CAR expression and CAR-T-cell therapy. cmRNA-based anti-CD19 CAR-T cells presented increased CAR intensity and prolonged duration of CAR expression in primary human T cells. Indeed, our results demonstrated that primary human T cells transfected with CAR-encoding cmRNAs were more effective at priming cytotoxic T cells than were those transfected with linear mRNAs in vitro. Compared with linear mRNAs, the intrinsic stability and long expression half-life of cmRNAs represent additional advantages in mRNA-based CAR-T-cell therapies, given that CAR-encoding mRNAs can be divided into daughter CAR-T cells and expanded CAR-T cells when they interact with targeted cells in vivo.

Scar sequences introduced during PIE-based circularization typically range from several dozen to a few hundred nucleotides in length. For example, the 186-nt scar from the Anabaena pretRNA group I intron has been reported to be able to induce innate immune activation. A recent study attempted to minimize potential immunogenicity by shortening the scar and utilizing the scar to achieve the desired pharmacological properties. The authors engineered the classical PIE system to shorten the scar to a 27-nt segment and leveraged the secondary structural features of the 27-nt scar to achieve the potent binding activity of the circular RNA aptamer.^40^ In this study, our scarless circularization platform completely avoided the introduction of the scar sequence. The removal of this extraneous sequence theoretically increases the overall coding capacity of the cmRNA, which will benefit the circularization of longer genes of interest. Across multiple in vitro and in vivo evaluations, including assessments of CAR expression and function as well as persistence and antitumor efficacy, scarred and scarless cmRNAs exhibited comparable CAR expression, cytotoxic activity, and no apparent differences in immunogenicity. Under multidose regimens, scarred and scarless cmRNA-based CAR-T cells achieved effective tumor control without significant differences. Compared with those in the scarred group, the antitumor activity of the scarless cmRNA-based CAR-T cells in the single-infusion group tended toward improved tumor suppression and longer survival. Together, these findings suggest that scarless cmRNAs may offer potential advantages, such as possibly reducing the risk of immunogenicity, improving RNA stability, and increasing the coding capacity for larger constructs, which also warrants further evaluation.

From a translational standpoint, the ability to generate functional CAR-T cells using cmRNAs offers practical advantages for manufacturing and regulatory review. Unlike integrating viral vectors, which necessitate extensive biosafety testing and carry a risk of insertional mutagenesis, cmRNA-based products can be produced rapidly, standardized, and scaled under chemically defined synthesis and purification conditions.^21,40^ Moreover, the transient expression profile of cmRNAs may lower the risk of prolonged or uncontrolled CAR activity, an attribute that is particularly pertinent to B cell–depleting applications in autoimmune disease, where a temporally bounded “immune reset” is desired.^17^ Finally, cmRNA‑programmed CAR T cells are amenable to repeat dosing and to combination strategies (for example, checkpoint blockade, cytokine support, or modulation of the tumor microenvironment), allowing efficacy to be augmented without permanent genetic modification of patient T cells.

Overall, we engineered a PIE platform to synthesize high-yield and scarless cmRNAs for potent CAR expression and long-lasting CAR-T-cell efficacy. Compared with their linear mRNA counterparts, cmRNA-based CAR-T cells elicited superior antitumor efficacy, as demonstrated by parallel lines of evidence, including in vitro specific cell killing, cytokine release, transcriptomic patterns, and in vivo tumor elimination and survival benefits. Our findings indicated that cmRNA-based CAR-T cells could efficiently eliminate target cells and provide long-lasting antitumor efficacy, providing a promising platform for mRNA-based CAR-T-cell therapies. Together with future advances in T-cell-targeted delivery systems, such as T-cell-specific lipid nanoparticles or antibody-guided carriers, cmRNAs will further unleash the full potential of mRNA technologies in CAR-based in vivo cell therapies.

## Materials and methods

### Cell culture and transfections

The HEK-293T, Raji, K562 and A549 cell lines were purchased from the Cell Bank, Chinese Academy of Sciences (CAS), and the NALM-6-luciferase (NALM-6-luc), MM.1S-luciferase (MM.1S-luc) and RPMI-8226-luciferase (RPMI-8226-luc) reporter cell lines were purchased from IMMOCELL. HEK-293T cells were cultured in DMEM (MeilunBio, MA0212) supplemented with 10% FBS (Cytiva, SH30396.03). Raji, K562, A549, NALM-6-luc, MM.1S-luc and RPMI-8226-luc cells were cultured in RPMI-1640 medium (MeilunBio, MA0215) supplemented with 10% FBS. Human primary PBMCs (peripheral blood mononuclear cells) were obtained from Hycells Company and maintained in RPMI-1640 with 10% FBS. Unless otherwise specified, all the cells were maintained at 37 °C in a humidified 5% CO_2_ atmosphere. For Gaussia luciferase (GLuc) and human erythropoietin (hEPO) expression, equimolar quantities of each RNA (equivalent to 100 ng) were transfected into HEK-293T cells per well in a 96-well plate via a Lipofectamine 3000 (Thermo Fisher Scientific, L3000001) according to the manufacturer’s instructions. For EGFP expression, equimolar quantities of RNA (equivalent to 500 ng) were transfected into HEK-293T cells per well in 24-well plates via a Lipofectamine 3000.

### Protein expression measurement

Luminescence from Gaussia luciferase was detected via a Pierce Gaussia Luciferase Glow Assay Kit (Thermo Fisher Scientific, 16161) on days 1, 2, 3, 4, 5, 6, and 7 after transfection. For luminescence detection at different time points, the cell culture medium was fully removed and replaced with fresh medium every day. For EGFP fluorescence detection, images were taken 24 h after transfection via an Olympus camera (CKX53). The cell culture supernatant was collected at 24 h after transfection and subjected to ELISA to measure hEPO expression via a Human Erythropoietin ELISA Kit (Abcam, ab274397).

### RT‒qPCR

Total RNA was extracted via TRIzol reagent (Invitrogen) following the manufacturer’s protocol. The RNA concentration and purity were assessed via a NanoDrop spectrophotometer (Thermo Fisher Scientific), with absorbance ratios of A260/A280 ≥ 1.8 and A260/A230 ≥ 2.0 indicating high-quality RNA. First-strand cDNA was synthesized from 1 μg of total RNA via a RevertAid First Strand cDNA Synthesis Kit (Thermo Scientific) with random hexamer primers. Reactions were performed under the following conditions: 25 °C for 5 min, 42 °C for 60 min, and 70 °C for 5 min. qPCR amplification was carried out via three-step reactions with SYBR^TM^ Green Master Mix (Thermo Fisher Scientific) on a QuantStudio 3 Real-Time PCR System (Applied Biosystems) following the manufacturer’s protocol. The primer pairs (Supplementary Table 3) were designed with Primer-BLAST to span exon‒exon junctions, and the amplification efficiency (90–110%) was validated via standard curves. Each 15 μL reaction contained 7.5 μL of SYBR^TM^ Green Master Mix, 1 μL of each primer (10 μM), 2 μL of cDNA template, and 3.5 μL of nuclease-free water. Relative gene expression was calculated via the 2^−ΔΔCt^ method, with GAPDH serving as the endogenous control. All reactions were performed in triplicate, and the data are presented as the means ± SDs.

### Vector construction and mRNA preparation

The protein coding sequence, IRES, 5’-UTR, 3’-UTR, and fragments of the group I intron were synthesized by TSINGKE and cloned and inserted into the pUC57 plasmid with a T7 promoter (Supplementary Table 1). The linearized plasmids were used as templates for in vitro transcription (IVT) via a T7 High Yield RNA Synthesis Kit (New England Biolabs, E2040). For the modified linear RNA, uridine was replaced with N1-methylpseudouridine (m1Ψ). The reactions were treated with DNaseI (New England Biolabs, M0303) after IVT. The synthesized RNA was column purified with a Monarch RNA Cleanup Kit (New England Biolabs, M2040). The linear RNA was capped with Cap 2’-O-methyltransferase (New England Biolabs, M0366) and the Vaccinia capping system (New England Biolabs, M2080), and the poly(A) tails were added via *E. coli* poly(A) polymerase (New England Biolabs, M0276) according to the manufacturer’s instructions.

### mRNA circularization and purification

The column-purified cmRNA precursors were circularized in T4 RNA ligase buffer (New England Biolabs, B0216) with 2 mM GTP at 55 °C for 15 min. Then, the products were treated with *E. coli* poly(A) polymerase (New England Biolabs, M0276) according to the manufacturer’s instructions and column purified. The purified RNA products were then treated with RNase R (Applied Biological Materials, E049) according to the manufacturer’s instructions and column purified to obtain enriched cmRNA. For cmRNA splice junction sequencing, RNase R-treated cmRNA was reverse transcribed into cDNA via a RevertAid First Strand cDNA Synthesis Kit (Thermo Fisher Scientific, K1622) with random primers. The PCR was performed with primers crossing the splice junction site, and the PCR products were sequenced by Sanger sequencing. cmRNAs were further analyzed via capillary electrophoresis on a QIAxcel Connect machine via the QIAxcel RNA High Sensitivity Cartridge Kit (QIAGEN, 929112).

### Lipid nanoparticle (LNP) formulation and characterization

The ionizable lipid DLin-MC3-DMA, along with DSPC, DMG-PEG 2000, and cholesterol, was sourced from AVT Pharmaceutical Technology Company. For the formulation of MC3-LNPs, a lipid mixture composed of 50 mol% DLin-MC3-DMA, 10 mol% DSPC, 38.5 mol% cholesterol, and 1.5 mol% DMG-PEG was prepared in ethanol. The RNA was suspended in a 50 mM sodium citrate buffer solution adjusted to pH 4.0. The LNP-mRNA complex was fabricated through the emulsion of the lipid organic phase and the RNA aqueous phase at a microfluidic laminar flow rate of 12 mL/min, with an aqueous-to-organic phase flow rate ratio of 3:1, utilizing a rapid nanomedicine system (INanoTM L). The emulsion subsequently underwent a dialysis process, which was carried out three times, each for a duration of 2 h at 4 °C, against a PBS buffer solution with a pH of 7.4, utilizing a Pur-A-LyzerTM Mega Dialysis Kit (Millipore Sigma, PURG60020). The resulting LNP formulation was concentrated via an Amicon Ultra 50 K MWCO centrifugal filter device (Merck Millipore, UFC905096) and subsequently passed through a 0.22-μm syringe filter (Millipore, SLGPR33RB) to ensure sterility and purity. The particle size distribution of the LNPs was ascertained via dynamic light scattering, as measured with a Zetasizer Nano ZS apparatus (Malvern Panalytical). The encapsulation efficiency of RNA was quantified via a Quant-it RiboGreen RNA Assay Kit (Thermo Fisher Scientific, R11490) following the protocol outlined by the manufacturer.

### Linear mRNA- or cmRNA-based CAR-T-cell production

Human PBMCs were cultured in RPMI-1640 supplemented with 10% FBS and activated with Dynabeads^TM^ Human T-Activator CD3/CD28 (Gibco, 11132D). One day post-activation, IL-2 (PEPROTECH, 200-02) was supplemented at a final concentration of 300 U/mL, and the T-cell density was maintained between 0.5 × 10^6^ and 1 × 10^6^ cells/mL. The beads were removed on day 3. Human T-cell pellets were collected and resuspended in the electroporation buffer Opti-MEM (Gibco, 31985062), and the cell concentration was adjusted to 8 × 10^7^ cells/ml. A total of 250 µL of the cell suspension was mixed with equimolar quantities of linear mRNA and circular mRNA (equivalent to 20 µg) carefully prior to transfection. The cell–mRNA mixture was transferred into a 4 mm electroporation cuvette (BTX), and electroporation was performed via the ECM2001+ system. Upon completion of electroporation, the cells were transferred to the prewarmed culture medium for further cultivation.

### Flow cytometric analysis

The dead cells were excluded via a Zombie Violet^TM^ Fixable Viability Kit (BioLegend, 423114) following the manufacturer’s guidelines. CAR expression was determined by incubating CAR-T cells with PE-labeled human CD19 (20-291) protein (ACRO, CD9-HP2H3) for 30 min at room temperature in FACS buffer. Peripheral blood was obtained from retro-orbital bleeding and stained for the presence of total human T cells (anti-CD3), CAR-T cells (CD19 protein), and memory T cells (anti-CD45RA (BioLegend, 304112) and anti-CD62L (BioLegend, 304822)). A single-cell suspension was prepared for analysis via a CytoFlex S flow cytometer (Beckman). More than 10,000 events were acquired and analyzed for each sample.

### In vitro cytotoxicity

The cytolytic activity of CAR-T cells was assessed on days 1 and 4 post-electroporation. For anti-CD19 CAR-T-cell evaluation, CD19-positive (NALM-6 and Raji) and CD19-negative (K562) human tumor cell lines were used as target cells. Untransfected, linear mRNA-transfected, or cmRNA-transfected anti-CD19 CAR-T cells were cocultured with target cells in flat-bottom 24-well plates at E:T ratios of 1:1, 2:1, and 5:1, respectively, for 16 h. Following incubation, the cells were harvested and stained with Zombie Violet™ viability dye and anti-CD3 and anti-CD19 antibodies. Target cell lysis was quantified by flow cytometry and calculated via the following formula: % lysis = 100 × [1 − (CD19⁺ fraction in the experimental group/CD19⁺ fraction in the control group)]. For anti-GPRC5D CAR-T-cell analysis, GPRC5D-positive (MM.1S and RPMI-8226) and GPRC5D-negative (K562) tumor cells were used as targets. Untransfected, linear mRNA-transfected, or cmRNA-transfected anti-GPRC5D CAR-T cells were cocultured under identical conditions. After 16 h, the samples were stained with Zombie Violet™, anti-CD3, and anti-GPRC5D antibodies and analyzed via flow cytometry. Tumor cell lysis was determined analogously as follows: % lysis = 100 × [1 − (GPRC5D⁺ fraction in the experimental group/GPRC5D⁺ fraction in the control group)]. Supernatants from cytotoxicity cocultures were collected and analyzed for IFN-γ, TNF-α and IL-2 levels via ELISA kits (Multi Sciences: IFN-γ, EK180-96; TNF-α, EK182-96; and IL-2, EK102) following the manufacturer’s protocols.

### Bulk RNA-seq experiments and bioinformatics analysis

Control T cells (untransfected), linear mRNA CAR-T cells or cmRNA CAR-T cells (*n* = 3) were harvested at days 2 and 4 after electroporation. Total RNA was subsequently extracted from the cell samples via TRIzol Reagent (Thermo Fisher Scientific, 15596026CN) to prepare RNA libraries via the VVAHTS® Universal V8 RNA-seq Library Prep Kit for Illumina (Vazyme, NR605-0). Sequencing was performed via the Illumina NovaSeq 6000 platform.

To perform the preprocessing of raw data, TrimGalore (version 0.4.5) was used to remove aptamers and low-quality bases from the raw RNAseq reads for each fastq file of each sample. Next, the trimmed reads were aligned to the reference genome (refdata-gex-GRCh38-2020-A) via STAR (Spliced Transcripts Alignment to a Reference, version 2.7.10b),^52^ considering splice alignment. Finally, the STAR-generated bam files were quantified via featureCounts (version 2.0.6)^53^ to count the mapped reads for each gene, which generated a read count matrix for all the samples over all the genes. Downstream differential gene expression (DEG) analysis was performed on the basis of the read count matrix via the DESeq2 R package (v1.16.1).^54^ To calculate the TPM expression matrix, the rnanorm (version 1.5.1) was used on the basis of the refdata-gex-GRCh38-2020-A reference genome file. The refdata-gex-GRCh38-2020-A reference file was obtained from https://www.10xgenomics.com/.

Differentially expressed genes between CAR-T cells and control T cells were selected on the basis of the following rules: absolute value of log2-transformed fold change (log2FC) ≥ 1.0 and FDR-adjusted *P* value (adjP) ≤ 0.05. DEG analysis was performed for multiple group comparisons: linear-vs-UTD, circular-vs-UTD, and circular-vs-linear at days 2 and 4. When cmCAR-T cells were directly compared with mCAR-T cells, the threshold of log2FC was set to 0.5 to capture more significantly regulated genes (FDR-adjusted *P* value (adjP) ≤ 0.05). Gene Ontology (GO) and pathway enrichment analyses were conducted via the Enrichr-KG webserver^55^ via the GO_Biological_Process_2021, KEGG_2021_Human, and Reactome_2022 databases. T-cell functional signature genes were collected from recent literature^47,56^ to cover T-cell activation, coinhibitory, T-cell exhaustion, and T-cell cytotoxicity gene signatures. The dot plots were created via the ggplot2 R package, and the heatmap was generated via the ComplexHeatmap R package.^57^ The expression of individual genes was plotted as bar graphs via GraphPad Prism.

### Tumor model and treatments

Procedures involving mice were performed in accordance with the standards of Fudan University and approved by the Animal Ethical Committee of the School of Pharmaceutical Sciences, Fudan University (ethical permit 2023-08-SY-ZXY-82). Male NCG mice (20 ± 2 g body weight) were purchased from GemPharmatech (Nanjing, China) and maintained under specific pathogen-free conditions. NALM-6-luc cells (1 × 10^6^ cells) and MM.1S cells (1 × 10^6^ cells) were intravenously injected into the mice on day 0. Tumor growth was monitored via a bioluminescence imaging (BLI) system, and tumor-bearing mice were randomized into the indicated groups (*n* = 5 per group) on day 3. Untransfected T cells, linear mRNA-CAR-T cells or cmRNA-CAR-T cells were intravenously injected into the corresponding mice (3 × 10^6^ cells per mouse) at days 3, 8, 13, and 18. The tumor burden was monitored twice a week via the BLI system according to the instructions, and the total or mean photon number intensity of luciferase was quantified.

### Statistical analysis

Statistical analysis was performed by using GraphPad Prism software. Student’s t test and two-way ANOVA were used to determine the significance. Asterisks denote statistically significant *p* values (*p < 0.05, **p < 0.01, ***p < 0.001, *****p < 0.0001), and ns indicates no statistical significance (*p* > 0.05). The data are presented as the means ± SDs unless otherwise indicated.

## Supplementary information

Supplementary information

Supplementary Table 1

Supplementary Table 2

Supplementary Table 3

## Data Availability

RNA-sequencing data are available from the Gene Expression Omnibus NCBI database under accession number GSE276719. All data supported in the conclusions are included in the manuscript and supplementary materials.

## Code availability

This paper does not report the original code. Any additional information required to reanalyze the data reported in this paper is available from the lead contact on request.
