# Supplementary material for: Scarless circular mRNA-based CAR-T cell therapy elicits superior antitumor efficacy
Source: Signal Transduct Target Ther. 2025 Dec 23;10:411. doi: 10.1038/s41392-025-02512-4 (PMC12722250; doi:10.1038/s41392-025-02512-4)
Supplement: Supplementary file 1 — Supplementary information [file 41392_2025_2512_MOESM1_ESM.docx]

Supplementary Materials for

**Scarless circular mRNA-based CAR-T-cell therapy elicits superior antitumor efficacy**

Qinchao Hu^1^, Hui Zhao^2^*, Kaicheng Zhou^1^, Xuefei Tian^1^, Qian Wang^1^, Xianxin Hua^3^*, Xuyao Zhang^1,4,5^*

Correspondence to: xuyaozhang@fudan.edu.cn

**This PDF file includes:**

Figures. S1 to S13

**Figure. S1.** **The synthesis and purification of CVB3-GLuc cmRNA**. **a** The real-scarless cmRNA of CVB3-GLuc (total 1299 nt in length) only includes CVB3 IRES (741 nt) and the coding sequencing of GLuc gene (558 nt). **b, c** Identification of CVB3-GLuc cmRNA using Poly(**a**) and RNase R treatments (**b**) and RNase H treatments (**c**). **d** Sanger sequencing of synthesized CVB3-GLuc cmRNA to confirm no extra sequences were introduced into cmRNA. **e, f** Capillary electrophoresis analysis to confirm efficient scarless (**e**) and scarred (**f**) circularization of CD19 CAR-encoding RNA. Lane 1: Size Marker; Lane 2: Linear CD19 CAR precursor mRNA control; Lane 3: Circularization reaction mixture containing CD19 CAR cmRNA and residual byproducts. **g** Purity of purified cmRNA using RNase R treatment was quantitatively measured by capillary electrophoresis.

**Figure. S2. Purified circular mRNA and chemically modified linear mRNA exhibit minimal innate immune activation in A549 cells**. (**a**-**e**) Relative mRNA expression levels of innate immune response genes (IL-6, DDX58, CCL5, TLR3, IFNB1) in A549 cells transfected with the following RNA formulations: Blank (Untransfected control); Circularization products (Crude RNA mixture containing cmRNA and byproducts); purified scarless cmRNA; purified scarred cmRNA; Linear mRNA (m1ψ, N1-methylpseudouridine-modified linear mRNA). RNA constructs were delivered into A549 cells via lipofectamine 3000 at 1 μg/10^6 cells. Total RNA was extracted 24 h post-transfection, and gene expression was analyzed by RT-qPCR using the ΔΔCT method with GAPDH as the endogenous control. Data are expressed as fold change relative to the Blank group. Data represent means ± SDs of triplicate biological replicates; statistical analysis by one-way ANOVA (****p<0.0001; ns, not significant).

**Figure. S3.** **Physicochemical Property Analysis of Lipid Nanoparticles**. **a, b** Conducting Zeta potential testing on the prepared Lipid Nanoparticles (LNP). **c, d** Compilation of a summary table for the detected Lipid Nanoparticles' (LNP) particle size, polydispersity index (PDI), and Zeta potential.

**Figure. S4. CAR-T cell yield and degradation profiles of cmRNA in T cells.** **a, b** Kinetics of absolute viable anti-CD19 (**a**) or anti-GPRC5D (**b**) CAR⁺ T cell numbers after mRNA electroporation (means ± SDs, n=3). **c, d** Degradation profiles of linear mRNA, scarred and scarless cmRNA in T cells. Electroporation of equimolar amounts of linear and circular mRNA into the same number of T cells (**c**). Detection of linear mRNA, scarred and scarless cmRNA by qPCR. Degradation kinetics of linear mRNA, scarred and scarless cmRNA within T cells over time (**d**). Data represent means ± SDs of triplicate biological replicates; statistical analysis by two-way ANOVA (*p < 0.05; **p < 0.01; ns, not significant).

**Figure. S5. Tumor cell line surface antigen expression identification. a** CD19 expression on B-cell leukemia/lymphoma lines (NALM-6, Raji) versus CD19-negative control (K562). **b** GPRC5D surface levels on multiple myeloma lines (MM.1S, RPMI-8226) versus GPRC5D-negative K562 cells.

**Figure. S6. cmCAR-T cells exhibit antigen-specific cytotoxicity without cytokine elevation against antigen-negative tumors**. **a** Cytotoxic activity of anti-CD19 CAR-T cells generated by linear mRNA, scarred cmRNA or scarless cmRNA platforms, assessed one- or four-days post-transfection against CD19-negative K562 cells at effector-to-target (E: T) ratios of 1:1, 2:1, and 5:1. **b** Cytotoxic activity of anti-GPRC5D CAR-T cells generated by linear mRNA, scarred cmRNA or scarless cmRNA platforms, assessed one- or three-days post-transfection against GPRC5D-negative K562 cells at effector-to-target (E: T) ratios of 1:1, 2:1, and 5:1. **c** Cytokine profiles (IFN-γ, TNF-α, IL-2) of anti-CD19 CAR-T cells 1-day post-transfection following antigen-independent stimulation with K562 cells. **d** Cytokine secretion by anti-GPRC5D CAR-T cells post-stimulation with antigen-negative targets. Data represent means ± SDs of triplicate biological replicates; statistical analysis by two-way ANOVA (ns, not significant).

**Figure. S7. Systematic profiling of immune checkpoint gene expression in CAR-T cell subgroups.** **a**-**h** The expression levels (TPM) of key co-inhibitory receptor genes (HAVCR2, BTLA, CD160, LAG3, CTLA4, PDCD1, KLRG1, CD244) were quantified across mCAR-T and cmCAR-T groups at day 2 and day 4 post-transfection. Data represent means ± SDs; n = 3; statistical analysis by two-way ANOVA (*p < 0.05; **p < 0.01; ***p < 0.001; ****p<0.0001; ns, not significant).

**Figure. S8. ssGSEA-based activation profiles of well-defined pathways and differential expression of representative genes**. **a** Heatmap showing relative expression levels of representative genes from well-defined pathways across samples. **b** ssGSEA enrichment scores of these pathways, together with KEGG, Reactome, and Hallmark gene sets, row-standardized to indicate relative activation levels across samples. **c** Expression differences of representative genes from the well-defined pathways. Differential expression was analyzed using DESeq2 (based on fold change and statistical significance), with group-wise significance indicated by annotations (*p < 0.05; ***p < 0.001; ****p < 0.0001; ns, not significant**)**

**Figure. S9.** **Flow cytometry gating strategy and** **flow cytometric data. a** gating strategy used for identifying and analyzing anti-CD19 CAR T-cell populations. **b** Individual flow cytometric data for mice presented in Fig. 7I of the manuscript. **c** gating strategy used for identifying and analyzing memory T-cell populations. **d** Individual flow cytometric data for mice presented in Fig. 7J of the manuscript.

**Figure. S10. Different cellular phenotypes of memory T cells. a** T_CM_ cells, CD45RO^+^CD45RA^−^ CD62L^+^; **b** T_EM_ cells, CD45RO^+^CD45RA^−^CD62L^−^; **c** T_EMRA_ cells, CD45RO^+^CD45RA^+^CD62L^−^.

**Figure. S11. Detection of circulating tumor cells in peripheral blood. a** Representative flow cytometry plots showing the presence of NALM-6 tumor cells (CD19^+^ human cells) in the peripheral blood of mice on day 20 post-tumor challenge. **b** Quantitative analysis of the percentage of circulating NALM-6 tumor cells in peripheral blood from each group. Data are presented as the means ± SEMs (n = 3 mice per group). Statistical significance was determined by one-way ANOVA (****P < 0.0001; ns, not significant).

**Figure. S12. Characterization of Lentivirus-transduced CAR-T cells and systemic inflammatory response post-infusion.** **a** Detection of lentivirus-transduced CAR-T cells using flow cytometry. **b** Serum levels of CRP on the third, seventh and twentieth days in the different groups after CAR-T cell transfer as measured using ELISA. (Data are presented as the means ± SDs; n=3; statistical analysis by one-way ANOVA; ns, not significant).

**Figure. S13. The antitumor efficacy of Linear mRNA-, Scarred cmRNA- or Scarless cmRNA-based CAR-T cells of single infusion. a** Schematic of the NALM-6 tumor model and single-dose treatment schedule. **b** Tumor burden was monitored by bioluminescence imaging analysis, n = 5 mice per group. **c, d** NALM-6 tumor burden was quantified as the total flux (**c**, each line represents single mouse) or mean flux (**d**) from luciferase intensity of each mouse. **The yellow-green dashed line represents the background value.**
